# Gasto catastrófico em crianças com deficiência visual: estudo transversal com cuidadores no Rio de Janeiro, Brasil

**DOI:** 10.1590/0102-311XPT167723

**Published:** 2024-11-11

**Authors:** Letícia Baptista de Paula Barros, Andrea Zin, Martha Cristina Nunes Moreira, Marisa da Silva Santos, Ana Carolina Carioca da Costa, Daniel de Souza Campos, Luiza M. Neves, Lorena Haefeli, Aline Piovezan Entringer, Maria Elisabeth Lopes Moreira, Fernanda Rebelo, Zilton Farias Meira de Vasconcelos, Márcia Pinto

**Affiliations:** 1 Instituto Nacional de Saúde da Mulher, da Criança e do Adolescente Fernandes Figueira, Fundação Oswaldo Cruz, Rio de Janeiro, Brasil.; 2 Instituto Nacional de Cardiologia, Rio de Janeiro, Brasil.; 3 Escola de Serviço Social, Universidade Federal do Rio de Janeiro, Rio de Janeiro, Brasil.; 4 Escola Nacional de Saúde Pública Sergio Arouca, Fundação Oswaldo Cruz, Rio de Janeiro, Brasil.

**Keywords:** Gasto Catastrófico, Cuidadores, Deficiência Visual, Cegueira, Baixa Visão, Catastrophic Expenditure, Caregivers, Visual Impairment, Blindness, Low Vision, Gasto Catastrófico, Cuidadores, Trastornos Visuales, Ceguera, Baja Visión

## Abstract

O cuidado de um filho com deficiência visual pode vir a afetar a renda do cuidador e, por sua vez, a renda da família. Sob essa realidade, há de se considerar o gasto catastrófico consequente do aumento de despesas e da redução de renda, seja pelo desemprego, pela redução do número de horas trabalhadas ou pela dificuldade de (re)inserção no mercado de trabalho. Perante esse cenário, o objetivo principal deste estudo foi estimar o gasto catastrófico atribuído ao cuidador de crianças cegas ou com baixa visão em centros de referência em educação para cegos, oftalmologia e saúde infantil localizados no Município do Rio de Janeiro, Brasil, identificando quais fatores estão associados a uma maior ou menor prevalência desse gasto. Observou-se que 53,3% dos cuidados de crianças com cegueira comprometem 40% ou mais da renda. Entre os cuidadores de crianças com baixa visão, o gasto catastrófico é mais ameno, comprometendo no mínimo 40% da renda para 36,8% dos cuidadores. Os fatores associados à maior prevalência de gasto catastrófico foram idade do cuidador, número de moradores na residência, maior escolaridade, menor renda domiciliar, reformas na residência, plano de saúde, aquisição de empréstimos, venda de bens, quantidade de unidades de saúde que a criança recebe tratamento e parentesco do cuidador principal. A carga que recai sobre os cuidadores de crianças com deficiência visual indica uma situação de vulnerabilidade que mostra a necessidade de acesso aos mecanismos de proteção financeira e social, por meio de políticas que sejam capazes de atender esse grupo.

## Abstract (en)

Caring for a visually impaired child can affect the caregiver’s income and, in turn, the family’s. Catastrophic spending resulting from increased expenses and reduced income must be taken into account, whether due to unemployment, a reduction in the number of hours worked or the difficulty of entering or reentering the job market. Given this scenario, the main objective of this study was to estimate the catastrophic spending attributed to the caregiver of blind or low-vision children in reference centers for education for the blind, ophthalmology and child health located in the city of Rio de Janeiro, Brazil, identifying which factors are associated with a higher or lower prevalence of this expenditure. It was found that 53.3% of care for blind children involved 40% or more of their income. Among the caregivers of children with low vision, catastrophic spending is milder, compromising at least 40% of income for 36.8% of the caregivers. The factors associated with a higher prevalence of catastrophic spending were the caregiver’s age, the number of residents in the household, higher schooling, lower household income, renovations to the home, health insurance, taking out loans, selling assets, the number of health units where the child receives treatment and the relationship of the main caregiver. The burden placed on caregivers of visually impaired children indicates a situation of vulnerability that shows the need for access to financial and social protection mechanisms, through policies that are capable of serving this group.

## Resumen

El cuidado de un niño con discapacidad visual puede impactar los ingresos del cuidador y, a su vez, de la familia. En este escenario, es necesario considerar el gasto catastrófico resultante del aumento de los gastos o la reducción de los ingresos, ya sea por desempleo, reducción del número de horas trabajadas o por la dificultad de inserción o reinserción en el mercado laboral. Ante esto, el objetivo principal de este estudio fue estimar el gasto catastrófico atribuido al cuidador de niños ciegos o con baja visión en centros de referencia en educación para ciegos, oftalmología y salud infantil, ubicados en el municipio de Río de Janeiro, Brasil, con el fin de identificar qué factores se asocian con una mayor o menor prevalencia de este gasto. Se observó que el 53,3% de los cuidados de niños con discapacidad visual comprometen más del 40% de los ingresos totales. Mientras tanto, el cuidado de niños con baja visión tiene un menor gasto catastrófico, comprometiendo menos del 40% de los ingresos según el 36,8% de los cuidadores. La mayor prevalencia de gasto catastrófico estuvo asociada a los siguientes factores: edad del cuidador, número de residentes en el hogar, mayor nivel de estudios, bajos ingresos familiares, remodelaciones en el hogar, seguro de salud, adquisición de préstamos, venta de bienes, cantidad de centros de salud en las que el niño acude al tratamiento y parentesco del cuidador principal. Los cuidadores de niños con discapacidad visual enfrentan una situación de vulnerabilidad, lo que apunta a una necesidad de acceder a acciones de protección financiera y social mediante políticas dirigidas a esta población.

## Introdução

Estimativas globais mostram que aproximadamente 2,2 bilhões de pessoas têm deficiência visual, sendo que metade desses casos poderiam ter sido evitados ou tratados ^1^. Entre 1990 e 2020, houve um aumento de 50,6% no número de pessoas cegas. Observou-se também um crescimento significativo de 91,7% para a deficiência visual moderada e grave, que atualmente acomete 295 milhões de pessoas ^2^ ^,^ ^3^.

A Organização Mundial da Saúde (OMS) calcula que cerca de metade das causas de deficiência visual infantil são preveníveis ou poderiam ser tratadas ^4^, como a retinopatia da prematuridade, a catarata e a opacidade da córnea ^5^. Em países de baixa e média renda, aproximadamente 4,8 milhões de crianças têm baixa visão, de um total estimado de 6 milhões em todo o mundo ^1^. No Brasil, as estimativas mostram que a deficiência visual atinge 18,8% da população, e a prevalência de cegueira infantil é de 0,5 a 0,6 por mil crianças ^6^.

A rotina diária dos cuidadores de crianças com deficiência visual pode incluir uma carga considerável de cuidado, como o acompanhamento à escola e aos serviços de saúde, além do cuidado e da realização das tarefas da casa. Essa rotina pode comprometer a atividade laboral quando tais cuidados exigem uma dedicação quase exclusiva, o que deriva em absenteísmo por necessidades das rotinas de cuidado e/ou redução do tempo de horas trabalhadas, ou ainda deixar o mercado de trabalho formal ^7^. Esse conjunto tão exaustivo de situações relacionadas ao cuidado de um filho/a com deficiência visual compromete o *status* de ocupação, podendo vir a afetar a renda do cuidador e, por sua vez, a da família. Sob essa realidade, há de se considerar o gasto catastrófico consequente do aumento de despesas e da redução de renda, seja pelo desemprego, pela redução do número de horas trabalhadas ou pela dificuldade de inserção ou de reinserção no mercado de trabalho ^8^ ^,^ ^9^ ^,^ ^10^ ^,^ ^11^.

Há uma variabilidade na escolha de limiares para se estimar o gasto catastrófico, como uma métrica de comparação entre renda e despesa do cuidador e da família devido às diferenças da estrutura de renda, dos mecanismos de proteção social, da condição clínica do paciente e dos sistemas de saúde ^9^ ^,^ ^12^ ^,^ ^13^. Em razão disso, encontram-se na literatura valores que podem alcançar até 40% na proporção da renda dos indivíduos ^8^ ^,^ ^14^. Ademais, há uma diversidade de termos para identificar o gasto, com a literatura trazendo alguns como o gasto com a alimentação ou gasto com subsistência, que está relacionado à participação específica desse gasto na renda em termos de percentis ^9^. O mesmo pode ser dito sobre a renda, que pode ser estimada com base em um escore de riqueza que mede as características dos municípios a partir de características do domicílio e dos bens ^15^.

Perante esse cenário, o objetivo principal deste estudo foi estimar o gasto catastrófico atribuído ao cuidador de crianças cegas ou com baixa visão em centros de referência em educação para cegos, oftalmologia e saúde infantil localizados no Município do Rio de Janeiro, Brasil. Além disso, buscou-se identificar quais fatores estão associados a uma maior ou menor prevalência desse tipo de gasto.

## Materiais e métodos

### Desenho do estudo e população

Trata-se de uma análise transversal utilizando dados de uma coorte mista multicêntrica denominada *Triagem de Doenças Oculares em Recém-nascidos e Perda de Qualidade de Vida por Cegueira ou Baixa Visão em Crianças: Uma Análise sob as Perspectivas Econômica e Social - Views-QoL Study* (https://linktr.ee/ViewsQOL). Foram selecionados cinco centros localizados no Município do Rio de Janeiro, especializados em educação para cegos, oftalmologia e saúde infantil e que recebem pacientes de diferentes regiões do Estado do Rio de Janeiro ^16^.

Neste artigo, foi feito um recorte do Views-QoL Study para estimar o gasto catastrófico entre os cuidadores informais de crianças com deficiência visual. Aplicou-se um questionário que incluiu variáveis sociodemográficas e econômicas. Um maior detalhamento dele pode ser encontrado em Moreira et al. ^16^.

O cuidador informal (nomeado no texto como “cuidador”) foi definido como o indivíduo responsável por prover cuidados sem receber nenhuma remuneração ou compensação financeira por esse trabalho ^17^.

Os participantes foram selecionados de acordo com o *status* da deficiência visual da criança e divididos em três grupos: cuidadores de crianças com cegueira; cuidadores de crianças com baixa visão; e cuidadores de crianças sem deficiência visual. Um indivíduo é considerado com baixa visão quando possui uma deficiência da função visual mesmo após o tratamento e/ou correção refrativa, mas usa sua visão ou é potencialmente capaz de usá-la para o planejamento e/ou execução de uma tarefa. Já um indivíduo é considerado cego se apresenta incapacidade total para ver e quando o prejuízo da visão alcança níveis incapacitantes para o exercício de tarefas rotineiras, apesar de apresentarem certos graus de visão residual ^6^.

### Treinamento para a coleta de dados

Os entrevistadores foram treinados durante para que se familiarizassem com o instrumento. Um projeto piloto com cinco entrevistas foi realizado previamente ao início da coleta com o intuito de avaliar as dúvidas e os problemas que poderiam surgir no decorrer do estudo. Esse treinamento também havia sido realizado para os entrevistadores do *Social and Economic Impacts of Zika Virus in Brazil* (estudo SEIZ; Impactos social e econômico do vírus Zika no Brasil) ^16^ ^,^ ^18^.

### Coleta de dados

A pesquisa foi realizada de forma presencial e individualizada enquanto o cuidador acompanhava a criança em um dos centros participantes. As entrevistas foram iniciadas em 2019 e foram paralisadas em março de 2020 devido à pandemia de SARS-CoV-2. Em agosto de 2020, houve a retomada do estudo, mas com o agravamento da pandemia foi encerrado em dezembro do mesmo ano. Para minimizar a perda da amostra devido à pandemia, foi necessário incluir 121 participantes do estudo SEIZ ^16^ ^,^ ^18^, conduzido entre 2017 e 2019. Essa pesquisa foi aninhada ao estudo de coorte *Vertical Exposure to Zika Virus and Its Consequences for Child Neurodevelopment: Cohort Study in Fiocruz/IFF* (Exposição vertical ao vírus Zika e suas consequências para o neurodesenvolvimento infantil: estudo de coorte no IFF/Fiocruz) (ClinicalTrials.gov, identifier NCT03255369), realizado em um hospital de referência nacional em saúde da criança. Daquele total, 26 casos de cuidadores de crianças com baixa visão e 95 cuidadores de crianças sem deficiência visual foram incorporados à amostra do Views-QoL Study. O estudo SEIZ também estimou o gasto catastrófico entre os cuidadores de crianças com síndrome congênita do vírus Zika e o mesmo instrumento foi utilizado no Views-QoL Study ^11^ ^,^ ^16^.

Foram considerados dois desfechos: gasto catastrófico acima de 10% e gasto catastrófico acima de 40%. As seguintes variáveis foram consideradas como sendo explicativas do desfecho: deficiência visual (cegueira e baixa visão); situação conjugal (com companheiro e sem companheiro); morar fora do Rio de Janeiro; principal cuidador (mãe, pai, avó e outro); idade do cuidador; moradores na residência; escolaridade (< 9 e ≥ 9 anos); renda domiciliar (classe A, classe B, classe C e classe D ou E); ocupação (deixou de trabalhar, manteve trabalho, continuou desempregado e começou a trabalhar); se recebe benefício governamental; mudança de local de residência; reformas na residência; cuidadores; hospitalizações; plano de saúde; empréstimos; quantidade de unidades de saúde em que a criança recebia o tratamento (1, 2 e ≥ 3); tempo de deslocamento do cuidador e da criança até a unidade de saúde (< 1, 1 ˫ 2, 2 ˫ 3 e ≥ 3 horas); e se precisou interromper os estudos após o nascimento.

### Esforço do cuidador

O “esforço do cuidador” foi representado pela quantidade de unidades de saúde que o cuidador frequentava periodicamente com a criança e pelo tempo de deslocamento entre elas e a residência. Essas poderiam fazer parte da rede de serviços do Sistema Único de Saúde (SUS) no município ou no estado do Rio de Janeiro ou serem particulares, além dos centros que oferecem assistência médica incluídos neste estudo. Para também expressar esse esforço, foi perguntado ao cuidador se ele havia parado ou interrompido os estudos após o nascimento da criança.

### Estimativa do gasto catastrófico

Neste estudo, a carga econômica do cuidador foi expressa por meio do gasto catastrófico, definido como o gasto do próprio bolso que excede determinada proporção da renda mensal média da família em razão da necessidade de arcar com os custos gerados por determinada doença. Foram assumidos limiares de 10% e 40% como proporção dessa renda, conforme consulta a literatura ^9^ ^,^ ^11^ ^,^ ^12^ ^,^ ^19^. Os resultados estão apresentados em reais (R$) de 2020.

Incorporou-se também a análise de coping costs, que é a busca de mecanismos de enfrentamento do cuidador e dos familiares da criança com deficiência visual (*coping strategies*) a fim de garantir a sobrevivência diária, como a venda de bens e o pedido de empréstimos ^11^ ^,^ ^19^ ^,^ ^20^.

Foi definida uma ampla gama de itens de custo para se estimar o gasto com o cuidado das crianças com deficiência visual, tendo como referência um instrumento previamente aplicado ^11^, por meio do qual os itens incluídos foram aqueles mencionados com mais frequência pelos cuidadores. Os seguintes itens foram considerados como despesas: alimentação na unidade de saúde e escola; fórmulas lácteas; medicamentos; exames; consultas; internações; óculos; lentes de contato; transporte utilizado na locomoção para as consultas; e exames e para o acompanhamento da criança em caso de internação. Incluiu-se também o pagamento de cuidadores para as outras crianças da casa, de plano de saúde para a criança com deficiência visual, além de despesas com mudança de local de residência e adaptações no domicílio com o objetivo de atender às necessidades específicas devido ao comprometimento da visão.

Para estimar a renda, aplicou-se o Critério Brasil (Associação Brasileira de Empresas de Pesquisa; http://www.abep.org/criterio-brasil), que estratifica os domicílios por classes de renda média mensal. As classes adotadas foram: classe A = R$ 22.749,24; classe B = R$ 8.255,14; classe C = R$ 2.544,64; classes D/E = R$ 862,41. Também foi perguntado ao cuidador se recebia algum tipo de benefício oriundo de programas de transferência de renda, como Benefício de Prestação Continuada (BPC) ou Bolsa Família/Auxílio Emergencial. Esses recursos foram excluídos da renda mensal pois não representam a renda do trabalho.

### Análise estatística dos dados

Os dados categóricos estão apresentados em tabelas a partir de suas frequências absolutas e relativas e os dados numéricos pela mediana e intervalo interquartil (IIQ). Quando a frequência absoluta foi menor do que cinco observações, as diferenças estatisticamente significativas foram avaliadas para as variáveis categóricas pelos testes qui-quadrado ou exato de Fisher. As variáveis numéricas foram avaliadas a partir do teste t de Student, e quando o pressuposto de normalidade for violado, utilizou-se o teste de Mann-Whitney. Foi realizada uma análise bivariada por meio do ajuste de um modelo de Poisson com variância robusta. As razões de prevalência foram apresentadas com seus respectivos intervalos de 95% de confiança (IC95%) para avaliar a força de associação das variáveis explicativas com o gasto catastrófico. Para todas as análises o nível de 5% de significância foi adotado para identificação de diferenças estatisticamente significativas. Utilizou-se o software IBM SPSS Statistics, versão 23 (https://www.ibm.com/).

### Considerações éticas

Esta pesquisa seguiu os critérios da *Resolução nº 466/2012* do Conselho Nacional de Saúde e foi aprovada pelos Comitês de Ética em Pesquisa dos cinco centros participantes (CAEE nº 06814819.2.3005.5246. O estudo SEIZ foi aprovado pelo Comitê de Ética em Pesquisa do Instituto Nacional de Saúde da Mulher, da Criança e do Adolescente Fernandes Figueira, Fundação Oswaldo Cruz (IFF/FIOCRUZ; CAEE nº 60682516.2.1001.5269). Os participantes assinaram o termo de consentimento livre e esclarecido e tiveram assegurado o direito de não responder a qualquer pergunta do questionário, bem como de desistir de participar da entrevista em qualquer momento sem prejuízo algum do seu tratamento.

## Resultados

### Características socioeconômicas e demográficas da amostra

O total da amostra foi de 355 cuidadores, dos quais 45 (13%) de crianças com cegueira, 133 (37%) de crianças com baixa visão e 177 (50%) de cuidadores de crianças sem deficiência visual. A maioria dos cuidadores eram mulheres-mães (91,5%), com idade entre 20 e 39 anos. Entre os cuidadores de crianças com cegueira, 11% eram as avós. O grupo responsável por crianças com cegueira tinha a maior proporção de cuidadores acima de 40 anos (29,5%). A maior parte dos cuidadores (52,4%) declarou residir no Município de Rio de Janeiro. Entre os grupos de cuidadores de crianças com cegueira e baixa visão, cerca de 56% em cada residiam fora da cidade. Para maiores detalhes, as características sociodemográficas e econômicas da amostra estão apresentadas em Moreira et al. ^16^.

### Esforço do cuidador

Entre os cuidadores de crianças que acessaram três ou mais unidades de saúde, 67,4% registraram gasto catastrófico para o limiar de 10% e 37,2% para o limiar de 40%, o que representou um indicativo de uma maior rotina de deslocamento (Tabela 1).

**Tabela 1:** Gasto catastrófico entre cuidadores de crianças com deficiência visual para os limiares de comprometimento de renda de 10% e 40%. Estado do Rio de Janeiro, Brasil, 2020.

Variáveis	10% da renda comprometida			40% da renda comprometida		
	n	%	Valor de p	n	%	Valor de p
Deficiência visual			0,054			0,052
Cegueira	41	91,1		24	53,3	
Baixa visão	104	78,2		49	36,8	
Situação conjugal			0,355			0,798
Com companheiro	100	83,3		50	41,7	
Sem companheiro	45	77,6		23	39,7	
Mora fora do Município do Rio de Janeiro	82	82,7	0,834	41	41	0,997
Principal cuidador			0,701			0,901
Mãe	130	80,7		41	41	
Pai	4	100,0		66	41	
Avó	9	81,8		1	25	
Outro	2	100,0		5	45,5	
Idade do cuidador [mediana (IIQ)]	32 (24; 40)		0,017	31 (27; 39)		0,931
Moradores na residência [mediana (IIQ)]	3 (2; 4)		0,342	3 (2; 4)		0,016
Escolaridade (anos)			0,464			0,048
< 9	31	77,5		11	27,5	
≥ 9	114	82,6		62	44,9	
Renda domiciliar *			0,092			0,034
Classe A	1	1,0		-	-	
Classe B	9	60,0		7	46,7	
Classe C	86	82,7		34	32,7	
Classes D ou E	49	84,5		32	55,2	
Ocupação			0,078			0,475
Deixou de trabalhar	65	87,8		35	47,3	
Manteve trabalho	36	75,0		18	37,5	
Continuou desempregado	15	65,2		7	30,4	
Começou a trabalhar	9	75,0		5	41,7	
Recebe benefício governamental	48	88,9	0,092	28	51,9	0,052
Despesas						
Mudança de local de residência	28	80,0	0,804	11	31,4	0,198
Reformas na residência	31	91,2	0,105	20	58,8	0,019
Cuidadores	7	87,5	0,653	1	12,5	0,093
Hospitalizações	47	79,7	0,664	26	44,1	0,559
Plano de saúde	35	100,0	0,002	33	94,3	< 0,001
Empréstimos	91	88,3	0,006	50	48,5	0,017
Quantidade de unidades de saúde em que a criança recebia o tratamento			0,019			0,031
1	68	84,0		27	33,3	
2	48	88,9		30	55,6	
≥ 3	29	67,4		16	37,2	
Tempo mediano de deslocamento do cuidador e da criança até a unidade de saúde (horas)			0,381			0,988
< 1	27	77,1		14	40,0	
1 ˫ 2	52	77,6		27	40,3	
2 ˫ 3	37	84,1		18	40,9	
≥ 3	29	90,6		14	43,8	
Precisou interromper os estudos após o nascimento	41	82,0	0,908	23	46,0	0,403

IIQ: intervalo interquartil.

* Faixas de renda média mensal, conforme o Critério Brasil: classe A = R$ 22.749,24; classe B = R$ 8.255,14; classe C = R$ 2.544,64; classes D e E = R$ 862,41.

### Estimativa do gasto catastrófico

A maioria dos cuidadores de crianças com cegueira (91,1%) e baixa visão (78,2%) registrou gasto catastrófico para o limiar de 10%. Considerando o limiar de 40%, o gasto catastrófico atingiu 53,3% dos cuidadores de crianças com cegueira e 36,8% dos cuidadores de crianças com baixa visão (Tabela 1).

Sob o limiar de 10%, as variáveis idade do cuidador, despesas com plano de saúde, pedido de empréstimo e número de unidades de saúde nas quais a criança recebia atendimento médico foram associadas de forma significativa ao gasto catastrófico. Para o limiar de 40%, as variáveis número de moradores na residência, escolaridade do cuidador, renda domiciliar, recebimento de benefício, despesas com plano de saúde, pedido de empréstimo, despesas com reforma na residência e quantidade de unidades de saúde que a criança recebia atendimento médico também se associaram fortemente ao gasto catastrófico (Tabela 1).

Entre os cuidadores de crianças com deficiência visual, para o limiar de 10%, o fato de o pai ser o cuidador principal indicou um aumento da prevalência de gasto catastrófico. Em relação à idade do cuidador, observou-se que quanto maior a idade, mais elevada foi a prevalência de gasto catastrófico (limiar de 10%). Os cuidadores de crianças com plano de saúde apresentaram maior prevalência de gasto catastrófico. Observou-se também para esse limiar que os cuidadores com renda média mensal da classe B apresentaram menor prevalência de gasto catastrófico em relação à classe de renda D/E (Tabela 2).

**Tabela 2:** Razões de prevalência de gasto catastrófico para os cuidadores de crianças com deficiência visual utilizando os limiares de 10% e 40% da renda familiar. Estado do Rio de Janeiro, Brasil, 2020.

Variáveis	10% da renda familiar			40% da renda familiar		
	RP	IC95%	Valor de p	RP	IC95%	Valor de p
Cuidador principal						
Mãe	Referência	Referência	-	Referência	Referência	-
Pai	1,258	1,163; 1,360	< 0,001	0,805	0,145; 4,464	0,804
Avó	1,029	0,771; 1,375	0,846	0,870	0,326; 2,368	0,797
Idade do cuidador	1,007	1,001; 1,013	0,016	0,995	0,972; 1,019	0,696
Renda domiciliar						
Classe A	-	-	-	-	-	-
Classe B	0,619	0,381; 1,004	0,052	0,234	0,063; 0,868	0,030
Classe C	0,937	0,815; 1,077	0,359	0,338	0,215; 0,532	< 0,001
Classe D ou E	Referência	Referência	-	Referência	Referência	-
Reformas na residência	1,077	0,916; 1,268	0,369	1,588	1,002; 2,518	0,049
Venda de bens	1,193	1,034; 1,377	0,016	1,612	0,922; 2,818	0,094
Plano de saúde	1,185	1,035; 1,357	0,014	1,260	0,768; 2,095	0,353
Pedido de empréstimo	1,274	1,079; 1,504	0,004	2,913	1,615; 5,254	< 0,001

IC95%: intervalo de 95% de confiança; RP: razão de prevalência.

Em relação ao limiar de 40%, houve aumento da prevalência do gasto catastrófico a partir da redução da faixa de renda. Não foram obtidos resultados para a classe de renda A, pois o número de famílias foi pequeno. A necessidade de reformas na residência foi significativa no aumento da prevalência de gasto catastrófico para o limiar de 40%, enquanto a contratação de plano de saúde elevou a prevalência de gasto catastrófico (limiar de 10%). Foi necessário lançar mão de *coping strategies* e a venda de bens foi um fator determinante para o gasto catastrófico (limiar de 10%). Independentemente do limiar adotado, o pedido de empréstimo foi uma estratégia entre os cuidadores, sendo esse fator também determinante para o aumento na prevalência (Tabela 2).

## Discussão

Neste estudo, foi possível estimar o gasto catastrófico atribuído ao cuidador de crianças com cegueira ou baixa visão recrutados em centros de referência do Município do Rio de Janeiro. Observou-se que 91,1% dos cuidadores comprometem no mínimo 10% da renda familiar com custos gerados pela deficiência visual da criança, sendo que mais da metade (53,3%) comprometem 40% ou mais da renda. Entre os cuidadores de crianças com baixa visão, o gasto catastrófico é mais reduzido, comprometendo no mínimo 10% da renda em 78,2% dos cuidadores e no mínimo 40% da renda em 36,8% dos cuidadores. Os fatores associados a maior prevalência de gasto catastrófico foram idade do cuidador, número de moradores na residência, maior escolaridade, menor renda domiciliar, reformas na residência, plano de saúde, aquisição de empréstimos, venda de bens, quantidade de unidades de saúde que a criança recebe tratamento e parentesco do cuidador principal.

O fato de a mãe não ser a cuidadora foi associado, no caso do limiar de 10%, ao aumento da prevalência de gasto catastrófico. Tais achados encontram correspondências com outras pesquisas, em que o cuidado às crianças e aos adolescentes com deficiência ou doenças raras são majoritariamente exercidos por mulheres, com alta carga social, afetiva e funcional ^11^.

Sob esse aspecto, cabe trazer a questão do papel das mulheres quando se discute o seu cotidiano, especialmente quando o cuidador é o responsável por uma criança com deficiência. Neste estudo, o conceito de cuidador se referiu ao responsável pela criança que não é remunerado pelas atividades de cuidado que exerce. O cuidado informal é uma atividade que estabelece uma triangulação entre o pouco reconhecimento, a feminilização e a economia do cuidado (*care economy*). Essa tríade gera desigualdades de gênero ao se verificar que as mulheres gastam de duas a dez vezes mais tempo com o cuidado informal do que os homens. Isso repercute na redução da participação da mulher na força de trabalho, na massa salarial e na qualidade do trabalho ^21^ ^,^ ^22^. Esse último aspecto pode levar ao *occupational downgrading*, no qual as mulheres acabam por aceitar uma ocupação que exige menor qualificação que a sua capacidade técnica e piores condições de trabalho ^21^. Nossos resultados não permitem especular se os cuidadores de crianças com deficiência visual que retornaram ao mercado de trabalho experimentaram tal realidade, sendo um tema importante de pesquisa futura a ser provocado.

O papel do cuidador é vital no dia a dia dos pacientes para prover cuidados físicos, funcionais e emocionais ^23^. E, apesar de sua importância, o cuidado informal não está comumente inserido na agenda de políticas, embora a carga econômica, emocional e a perda de qualidade de vida sejam substanciais ^11^ ^,^ ^19^ ^,^ ^20^ ^,^ ^24^. Especificamente para a carga econômica, um estudo recente mostrou que o custo por perda de produtividade de cuidadores de adultos com doenças cardiovasculares, respiratórias e câncer no Brasil foi de cerca de US$ 18 bilhões ao ano (R$ 90 bilhões), ou 1,38% do produto interno bruto (PIB) de 2020 ^25^. Portanto, sugere-se ampliar as pesquisas que tragam como objeto a economia do cuidado e suas repercussões econômicas e sociais para o país.

Expressamos aqui a carga econômica por meio do gasto catastrófico. Para a métrica dele, em geral, não há limiares estabelecidos na maioria dos países ^8^ ^,^ ^14^, diante de diferenças na estrutura da renda de cada indivíduo e da família e do sistema de proteção social. Alguns estudos estimaram o gasto catastrófico (às vezes de forma arbitrária) com pontos de corte que variaram sobremaneira ^9^ ^,^ ^12^ ^,^ ^13^. No caso desse estudo, os limiares também foram selecionados arbitrariamente conforme estudo prévio ^11^.

A literatura tem demonstrado que a presença de condições crônicas em crianças aumenta essa carga sobre as famílias e os cuidadores ^11^ ^,^ ^19^ ^,^ ^26^ ^,^ ^27^ e, como aqui observado, a deficiência visual também está associada com uma elevada prevalência de gasto catastrófico. Nesse sentido, podemos trazer o conceito de *financial toxicity*, que toca nos objetivos e resultados deste estudo e tem sido aplicado à vivência cotidiana de pacientes oncológicos e seus cuidadores ^28^, mas que, em nossa opinião, coaduna-se com outras condições de saúde. Há duas formas de *financial toxicity*: objetiva (relacionada aos gastos do próprio bolso) e subjetiva (considera o estresse percebido devido a tais gastos) ^28^. A primeira se aproxima do que se observou em nossos resultados, pois houve gasto catastrófico para o grupo de cuidadores de crianças com deficiência visual. Essa situação se revela ainda mais preocupante quando se verificou que mais de 30% desses cuidadores tinham renda média mensal menor que um salário mínimo vigente na época do estudo ^16^, guiada por uma constatação de quanto menor a renda, maior foi a prevalência de gasto catastrófico.

Os programas de transferência de renda podem minimizar a vulnerabilidade econômica da criança e sua família com deficiência visual. Mesmo com os cuidadores dessas crianças tendo acesso a algum benefício oriundo de programas de transferência de renda, observou-se que o gasto catastrófico ainda estava presente para cerca de 90% (limiar de 10%) e 52% (limiar de 40%). Nesse sentido, opera como uma barreira ao direito das pessoas com deficiência critérios como o limite da renda por pessoa do grupo familiar e as avaliações médica e social. Ademais, isso contribui para que as mulheres-mães não possam estar no mercado de trabalho, tornando a unidade familiar dependente do benefício recebido.

O pedido de empréstimo foi prevalente para os cuidadores de crianças com deficiência visual, resultado similar ao encontrado em outros estudos nos quais foi necessário acessar coping strategies para cobrir gastos extras com a condição de saúde da criança ^11^ ^,^ ^19^ ^,^ ^20^. Essa realidade reforça o que outras pesquisas têm apontado como necessidades não atendidas dos cuidadores de pacientes pediátricos no Município do Rio de Janeiro, referindo-se à ampliação de mecanismos de proteção financeira para famílias de crianças com condições crônicas complexas ^11^ ^,^ ^19^.

Embora nesta pesquisa a escolaridade do cuidador não tenha sido associada à condição de visão da criança, os resultados indicaram que uma menor escolaridade esteve associada ao gasto catastrófico (limiar de 40%). Isso pode reforçar a importância das políticas sociais que reconheçam o lugar das mulheres no trabalho de cuidado e em suas múltiplas jornadas e das de investimento em escolarização ^29^. Observou-se que, mesmo que os resultados não tenham sido significativos, naquelas famílias em que o cuidador precisou interromper os estudos, 46% apresentaram gasto catastrófico (limiar de 40%). Essa realidade vem sendo apresentada na literatura diante de cuidadores que interrompem seus estudos, podendo ampliar as desigualdades e o desafio dos deles de aprenderem sobre a doença e compreenderem o processo terapêutico da criança ^30^ ^,^ ^31^. Se isso opera para mães/mulheres dedicadas às crianças típicas/sem deficiência, cabe refletir o quanto o trabalho de cuidado muitas vezes significa abandono de projetos pessoais, como escolaridade e carreira, quando as redes de apoio se ausentam ou enfraquecem.

A expansão das redes de vizinhança e amizade também pode contribuir com a produção de capital social, não se restringindo somente à família de parentesco, em uma leitura que aponta para a capacidade de agência dos indivíduos ^32^. Em que pese uma perspectiva fortemente neoliberal nessa discussão, ainda assim cabe revê-la à luz das iniquidades e das desigualdades sociais que desfavorecem determinados grupos populacionais, com destaque para as mulheres negras, pobres, periféricas, chefes de família, e com filhos com deficiências e/ou condições de saúde raras, complexas e crônicas ^33^. Nesse sentido, a organização da rede de serviços de saúde, educacionais e de assistência social para facilitar o dia a dia dos cuidadores é mais que urgente, diante de necessidades específicas desses grupos que inclusive refletem na efetividade do tratamento das crianças. Sugere-se, diante da sua relevância, que se amplie as pesquisas no Brasil nesse tema para nortear direcionamentos de diferentes ordens e que sejam possíveis e efetivos para essas pessoas.

Os cuidadores de crianças com cegueira frequentaram mais unidades de saúde durante o acompanhamento dela no tratamento do que aqueles de crianças com baixa visão. Ressalta-se que mais de 80% dos cuidadores nos quais houve presença de gasto catastrófico para o limiar de 10% acessavam somente uma unidade de saúde. Podemos discutir que, apesar de terem mencionado essa única unidade de tratamento, isso pode estar relacionado com o fato de existir alguma restrição financeira e também de tempo por parte do cuidador que o impede de acessar outros serviços de saúde. Ou seja, pode não significar que a criança tenha acesso a todos os tipos de serviços que necessita na unidade de saúde, mas sim uma questão de escolha do cuidador do local mais acessível. Acredita-se que esse “ir e vir” impõe maiores gastos, além do cansaço físico vivenciado na rotina de espera para atendimento médico e de utilização do sistema de transporte municipal e intermunicipal ^34^. Ainda que se tenha modais no Município do Rio de Janeiro, como barcas, ônibus, trens e metrôs, não significa necessariamente que sejam facilmente acessíveis aos cuidadores de outras regiões do estado, considerando que mais da metade dos cuidadores de crianças com deficiência visual moravam fora do Município do Rio de Janeiro.

Este estudo aplicou um instrumento semelhante ao utilizado por Pinto et al., que estimou o gasto catastrófico de cuidadores de crianças com a síndrome congênita do vírus da zika ^11^ e de crianças com doenças raras ^19^. Porém, há diferenças na natureza das condições estudadas que contribuem para a distinção entre os mesmos, pois o estudo atual considerou as famílias de crianças com cegueira ou baixa visão causada por uma variedade de outros diagnósticos. Sob esse aspecto, diferentes diagnósticos podem acarretar necessidades e desafios específicos para as famílias envolvidas, exigindo abordagens de intervenção e suporte distintas. Nesse sentido, estimar o gasto catastrófico no campo da deficiência visual pode começar a preencher uma lacuna na literatura de estudos semelhantes. Até onde sabemos, são escassas as pesquisas dessa natureza no Brasil, e sua originalidade pode contribuir para fornecer mais evidências para as políticas e as práticas destinadas à ampliação de mecanismos de proteção financeira aos cuidadores.

Este estudo tem algumas limitações. A pandemia de SARS-CoV-2 iniciada em 2020 prejudicou o cronograma do Views-QoL-Study, sendo necessário acessar alternativas, ainda que não menos robustas, para se obter os resultados esperados. A inclusão da amostra de cuidadores de crianças com baixa visão do estudo SEIZ foi necessária devido à perda de casos a partir da pandemia de COVID-19 em março de 2020. No entanto, para minimizar um possível viés de seleção, foram selecionados cuidadores de crianças com diagnóstico clínico confirmado de deficiência visual que atendiam aos critérios de inclusão do Views-QoL-Study. A ocorrência de viés de informação (memória) na aplicação dos questionários também deve ser considerada. Diferentes variáveis relacionadas à rotina das crianças e que afetariam o montante do gasto não foram exploradas no estudo, como por exemplo outras deficiências. Nesse sentido, sugerimos que mais pesquisas sejam realizadas e que o escopo seja ampliado com o objetivo de identificar outros determinantes do gasto catastrófico. A generalização dos resultados apresentados é limitada devido à realização do estudo somente em centros localizados no Município do Rio de Janeiro, podendo não representar a diversidade da realidade brasileira. Finalmente, a variável raça não foi considerada no questionário.

## Conclusão

A presença da deficiência visual em crianças está associada ao incremento da carga econômica e às exigências de cuidado que recaem grandemente sobre as mulheres-mães. Tais cargas materiais - organização de rotinas, planejamento de orçamento, luta por direitos não contemplados, interrupção de carreira, investimento em escolaridade, trabalho formal, cuidados com a própria saúde - podem se associar a quadros de necessidades não atendidas, dignos de atenção para futuras pesquisas.

Cuidadores de crianças com deficiência visual, em sua maioria, precisam abdicar de seus trabalhos para viverem exclusivamente se dedicando aos cuidados da criança. Tal retrato indica a importância de uma abordagem que inclua as mulheres-mães em ações de suporte social e saúde mental, além de políticas de renda que contemplem cuidadores domiciliares remunerados. Além disso, cabe refletir sobre a necessidade de criação de centros que ofereçam uma oferta de diferentes serviços, minimizando o desgaste e gastos com o deslocamento.

## Data Availability

Os dados da pesquisa foram armazenados na plataforma FormSUS (http://siteformsus.datasus.gov.br/FORMSUS/index.php).

## References

[1] World Health Organization Vision impairment and blindness..

[2] Vos T, Lim SS, Abbafati C, Abbas KM, Abbasi M, Abbasifard M (2020). Global burden of 369 diseases and injuries in 204 countries and
territories, 1990-2019: a systematic analysis for the Global Burden of
Disease Study 2019.. Lancet.

[3] Bourne R, Steinmetz JD, Flaxman S, Briant PS, Taylor HR, Resnikoff S (2021). Trends in prevalence of blindness and distance and near vision
impairment over 30 years an analysis for the Global Burden of Disease
Study. Lancet Glob Health.

[4] World Health Organization World report on vision..

[5] Gilbert C, Bowman R, Malik AN (2017). The epidemiology of blindness in children changing
priorities. Community Eye Health.

[6] Ottaiano JAA, de Ávila MP, Umbelino CC, Taleb AC As condições de saúde ocular no Brasil..

[7] Marques AP, Ramke J, Cairns J, Butt T, Zhang JH, Muirhead D (2021). Global economic productivity losses from vision impairment and
blindness. EClinicalMedicine.

[8] Flores G, Krishnakumar J, O'Donnell O, van Doorslaer E (2008). Coping with health-care costs: implications for the measurement
of catastrophic expenditures and poverty.. Health Econ.

[9] Xu K, Evans DB, Kawabata K, Zeramdini R, Klavus J, Murray CJL (2003). Household catastrophic health expenditure a multicountry
analysis. Lancet.

[10] Pal R (2009). Analysing catastrophic OOP health expenditure in India: concepts,
determinants and policy implications.. SSRN.

[11] Pinto M, Moreira MEL, Barros LBP, Costa ACC, Fernandes S, Kuper H (2021). Gasto catastrófico na síndrome congênita do vírus Zika:
resultados de um estudo transversal com cuidadores de crianças no Rio de
Janeiro, Brasil.. Cad Saúde Pública.

[12] Boing AC, Bertoldi AD, Barros AJD, Posenato LG, Peres KG (2014). Desigualdade socioeconômica nos gastos catastróficos em saúde no
Brasil. Rev Saúde Pública.

[13] Lu L, Jiang Q, Hong J, Jin X, Gao Q, Bang H (2020). Catastrophic costs of tuberculosis care in a population with
internal migrants in China. BMC Health Serv Res.

[14] Shrime MG, Dare AJ, Alkire BC, O'Neill K, Meara JG (2015). Catastrophic expenditure to pay for surgery worldwide: a
modelling study.. Lancet Glob Health.

[15] Macedo J (2020). Gastos catastróficos em saúde na população de Minas Gerais: tendências e
associação com condições sociodemográficas entre 2009 e 2013.

[16] Moreira MCN, Steffen RE, Zin AA, Santos MS, Costa ACC, Campos DS (2023). Depressão, ansiedade, estresse e apoio social estudo transversal
com cuidadores de crianças com deficiência visual no Rio de Janeiro, Brasil
- Views-QoL Study. Cad Saúde Pública.

[17] Koopmanschap MA, van Exel JNA, van den Berg B.Brouwer WBF (2008). An overview of methods and applications to value informal care in
economic evaluations of healthcare. Pharmacoeconomics.

[18] Kuper H, Lyra TM, Moreira MEL, de Albuquerque MSV, de Araújo TVB, Fernandes S (2018). Social and economic impacts of congenital Zika syndrome in Brazil
Study protocol and rationale for a mixed-methods study. Wellcome Open Res.

[19] Pinto M, Madureira A, Barros LBP, Nascimento M, Costa ACC, Oliveira NV (2019). Cuidado complexo, custo elevado e perda de renda o que não é raro
para as famílias de crianças e adolescentes com condições de saúde
raras. Cad Saúde Pública.

[20] Katumba KR, Tann CJ, Webb EL, Tenywa P, Nampijja M, Seeley J (2023). The economic burden incurred by families caring for a young child
with developmental disability in Uganda. PLOS Glob Public Health.

[21] Ferrant G, Pesando LM, Nowacka K Unpaid care work: the missing link in the analysis of gender gaps in
labour outcomes..

[22] Comisión Económica para América Latina Cuidados y mujeres en tiempos de COVID-19: la experiencia en la
Argentina..

[23] Pinto RA, Holanda MA, Medeiros MMC, Mota RMS, Pereira EDB (2007). Assessment of the burden of caregiving for patients with chronic
obstructive pulmonary disease. Respir Med.

[24] Kuhlthau K, Kahn R, Hill KS, Gnanasekaran S, Ettner SL (2010). The well-being of parental caregivers of children with activity
limitations. Matern Child Health J.

[25] Espinola N, Pichon-Riviere A, Casarini A, Alcaraz A, Bardach A, Williams C (2023). Making visible the cost of informal caregivers' time in Latin
America a case study for major cardiovascular, cancer and respiratory
diseases in eight countries. BMC Public Health.

[26] Zan H, Scharff RL (2015). The heterogeneity in financial and time burden of caregiving to
children with chronic conditions. Matern Child Health J.

[27] Belza C, Cohen E, Orkin J, Fayed N, Major N, Quartarone S (2023). Out-of-pocket expenses reported by families of children with
medical complexity. Paediatr Child Health.

[28] Pangestu S, Rencz F (2023). Comprehensive score for financial toxicity and health-related
quality of life in patients with cancer and survivors a systematic review
and meta-analysis. Value Health.

[29] Longo FV, Vieira JM (2017). Educação de mãe para filho fatores associados à mobilidade no
Brasil. Educação & Sociedade.

[30] Zhu Y, Gao J, Li X, Yang Q, Lian Y, Xiao H (2019). Burden, positive aspects, and predictive variables of caregiving
a study of caregivers of patients with pediatric glaucoma. J Ophthalmol.

[31] Kantipuly A, Pillai MR, Shroff S, Khatiwala R, Raman GV, Krishnadas SR (2019). Caregiver burden in primary congenital glaucoma. Am J Ophthalmol.

[32] Jordan J, Linden MA "It's like a problem that doesn't exist": the emotional
well-being of mothers caring for a child with brain injury (2013). Brain. Inj.

[33] Rodrigues MP (2021). Iniquidades raciais em saúde no Brasil uma revisão
integrativa. Revista da ABPN.

[34] Peiter P, Pereira R, França I (2020). Análise de dimensões do acesso à saúde das crianças com síndrome
congênita de Zika (SCZ) na Região Metropolitana do Rio de
Janeiro. Saúde Soc.

